# An Unusual Case of Aplastic Anemia Caused by Temozolomide

**DOI:** 10.1155/2010/975039

**Published:** 2010-12-01

**Authors:** Gazi Comez, Alper Sevinc, Ozlem Nuray Sever, Taner Babacan, Ibrahim Sarı, Celalettin Camci

**Affiliations:** ^1^Department of Internal Medicine, Faculty of Medicine, University of Gaziantep, 27310 Gaziantep, Turkey; ^2^Department of Medical Oncology, Faculty of Medicine, University of Gaziantep, 27310 Gaziantep, Turkey; ^3^Department of Pathology, Faculty of Medicine, University of Gaziantep, 27310 Gaziantep, Turkey

## Abstract

Radiotherapy and concomitant/adjuvant therapy with temozolomide are a common treatment regimen for children and adults with high-grade glioma. Although temozolomide is generally safe, it can rarely cause life-threatening complications. Here we report a case of a 31-year-old female patient who underwent surgical resection followed by radiotherapy plus concomitant temozolomide. She developed pancytopenia after adjuvant treatment with temozolomide. A bone marrow aspiration and biopsy showed hypocellularity with very few erythroid and myeloid cells, consistent with aplastic anemia. In the English literature, aplastic anemia due to temozolomide is extremely rare.

## 1. Introduction

The standard management of patients with glioblastoma multiforme (GBM) includes surgical resection followed by radiotherapy (RT) plus concomitant and adjuvant temozolomide [1]. Temozolomide (TMZ) is an alkylating agent and is generally well-tolerated treatment with fatigue, thromboembolic events, and lymphopenia as the most common side effects. Grade III or IV myelosuppression is a relatively uncommon side effect that is reported in 4% of patients [2]. We hereby describe a case of GBM treated with surgical resection followed by RT plus concomitant and adjuvant temozolomide, who developed a progressive irreversible aplastic anemia.

## 2. Case Report

**Figure 1:**
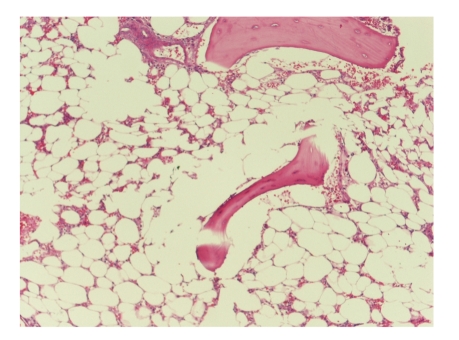
Severely hypocellular bone marrrow with very few erythroid and myeloid cells at any stage of differentiation suggesting features of aplastic anemia.

A 31-year-old female was admitted to the neurosurgery department with fainting and headache. MRI of the brain revealed a mass in the left frontal lobe. She underwent a left frontal craniotomy and complete excision revealing a glioblastoma multiforme (GBM). Later, she was started on antiepileptic medication, phenytoin, and referred for adjuvant treatment. She started concomitant brain RT with TMZ, administered at 150 mg/m^2^ for 5 days every 4 weeks. After three cycles of adjuvant TMZ therapy, she was admitted to the medical oncology department with fever, and complete blood count showed pancytopenia with hemoglobin level 6.3 g/dL (13.6–17.2), platelet count 19,000/mm^3^ (156,000–373,000),  and total leucocyte count 100/mm^3^. TMZ was stopped. She was treated with blood products, growth factors, and antibiotics. Phenytoin was changed to carbamazepine. Her medical history revealed a fever continuing for 30 days with productive cough. Chest radiograph and CT scan revealed an infiltration at the superior segment of the left lower lobe with pleural effusion. Aspergillus sp. grew in bronchial washing and sputum cultures, cytology revealed no malignancy, and cultures were negative for tuberculosis. Forty days later, blood tests showed hemoglobin level 8.3 g/dL (13.6–17.2), platelet count 16,000/mm^3^ (156,000–373,000) and total leucocyte count: 50/mm^3^. Clinical and radiographic findings were not improved. Bone marrow aspiration and biopsy were subsequently performed. The biopsy was severely hypocellular with very few erythroid and myeloid cells at any stage of differentiation and hardly any identifiable progenitor cells, suggesting features of aplastic anemia (Figure 1). Cytogenetic analysis of marrow was not performed. She did not receive co-trimoxazole prophylaxis, and her vitamin B12 and folic acid levels were normal. The patient had a rapidly deteriorating course and died due to septicemia.

## 3. Discussion

Adverse effects of TMZ are typical of traditional cytotoxic chemotherapy. Myelosuppression is often dose limiting with specific guidelines regarding dose reductions based on neutrophil and platelet counts. Myelosuppression tends to occur later in the treatment cycle; it is noncumulative and generally reversible within two weeks [3]. Grade 3 and 4 hematological toxicity was documented in only 7% of patients on concurrent therapy treated prospectively in the European Organization for Research and Treatment of Cancer (EORTC) and National Cancer Institute of Canada (NCIC) landmark study [2]. Gerber et al. described 52 patients developing severe myelosuppression after radiotherapy and concomitant adjuvant temozolomide therapy with high-grade gliomas [4]. 

 Aplastic anemia is classified as inherited or acquired depending on etiology, and nonsevere or very severe on the basis of degree of pancytopenia. Aplastic anemia is thought to result from injury or destruction of pluripotential stem cells affecting all subsequent blood cell populations. Erythrocytes, granulocytes, and platelets may decrease to dangerously low levels. The pathophysiology of aplastic anemia is now believed to be immune mediated, with active destruction of blood-forming cells. Drugs and chemicals are among the most frequent causes triggering the aberrant immune response [5].

 In the English literature, Doyle et al. described three patients developing severe myelosuppression after low-dose TMZ and RT. Two patients with aplastic anemia were given concurrent co-trimoxazole [6]. Villano et al. described a case of aplastic anemia after the 4th cycle of adjuvant TMZ, and the patient was on concomitant anticonvulsant [7]. In the case described here, the patient was also receiving phenytoin, which typically causes agranulocytosis rather than pancytopenia. Phenytoin was stopped but pancytopenia did not recover. 

 It is important to be aware of the potentially life-threatening toxicity of every chemotherapeutic agent, including temozolomide. Temozolomide should be prescribed with recognition of the potential side effects reported here.
